# Radiographic and fluoroscopic X‐ray systems: Quality control of the X‐ray tube and automatic exposure control using theoretical spectra to determine air kerma and dose to a homogenous phantom

**DOI:** 10.1002/acm2.13329

**Published:** 2021-07-01

**Authors:** Bente Konst, Jacob Nøtthellen, Ellinor Bilet, Magnus Båth

**Affiliations:** ^1^ Department of Radiology Vestfold Hospital Trust Tønsberg Norway; ^2^ Faculty of Mathematics and Natural Sciences Department of Physics University of Oslo Oslo Norway; ^3^ Division of Diagnostics and Intervention Oslo University Hospital Oslo Norway; ^4^ Norwegian Hospital Construction Agency Trondheim Norway; ^5^ Department of Medical Physics and Biomedical Engineering Sahlgrenska University Hospital Gothenburg Sweden; ^6^ Department of Radiation Physics Institute of Clinical Sciences Sahlgrenska Academy at University of Gothenburg Gothenburg Sweden

**Keywords:** absorbed dose, air kerma, automatic exposure control, fluoroscopy, quality control, radiography, X‐ray tube

## Abstract

**Purpose:**

To develop a method to perform quality control (QC) of X‐ray tubes and automatic exposure control (AEC) as a part of the QC of the radiographic and fluoroscopic X‐ray system. Our aim is to verify the output from the X‐ray tube by comparing the measured radiation output, or air kerma, to the theoretical output given the applied exposure settings and geometry, in addition to comparing the measured kV to the nominal kV. The AEC system for fluoroscopic and conventional X‐ray systems is assessed by determining the absorbed dose to a homogenous phantom with different thicknesses.

**Method:**

This study presents a model to verify the X‐ray tube measurement results and a method to determine the dose to a homogenous phantom (D_phantom_). The following input is needed: a parameterized model of the X‐ray spectrum, the X‐ray tube measurements using a multifunctional X‐ray meter, the exposure parameters recorded via imaging of polymethyl methacrylate (PMMA) slabs of different thickness that simulate the patient using AEC, and a parameterized model for calculating the dose to water from Monte Carlo simulations. The output is the entrance surface dose (ESD) and absorbed dose in the phantom, D_phantom_ (µGy). In addition, the parameterized X‐ray spectrum is used to compare theoretical and measured air kerma as a part of the QC of the X‐ray tube. To verify the proposed method, the X‐ray spectrum provided in this study, SPECTRUM, was compared to two commercially available spectra, SpekCalc and Institute of Physics and Engineering in Medicine (IPEM) 78. The fraction of energy imparted to the homogenous phantom was compared to the imparted fraction calculated by PCXMC.

**Results:**

The spectrum provided in this study was in good agreement with two previously published X‐ray spectra. The absolute percentage differences of the spectra varied from 0.05% to 3.9%, with an average of 1.4%, compared to SpekCalc. Similarly, the deviation from IPEM report 78 varied from 0.02% to 2.3%, with an average of 0.74%. The SPECTRUM was parameterized for calculation of the imparted fraction for target angles of 10°, 12°, and 15°, kV (50–150 kV) with the materials Al (2.2–8 mm), Cu (0–1 mm), and any combination of the filters, PMMA and water. The deviation of energy imparted from the results by PCXMC was less than 8% for all measurements across different kV, filtration, and vendors, obtained by using PMMA to record the exposure parameters, while the dose was calculated based on water with same thicknesses as the PMMA.

**Conclusion:**

This study presents an accurate and suitable method to perform a part of the QC of fluoroscopic and conventional X‐ray systems with respect to the X‐ray tube and the associated AEC system. The method is suitable for comparing protocols within and between systems via the absorbed dose.

## 1 INTRODUCTION

The complexity of medical X‐ray procedures is steadily increasing and the sophistication of radiology enables more advanced procedures, which has expanded the range of applications. The filtration and X‐ray beam qualities vary, leading to changing corresponding dose ranges. According to IAEA, there are two reasons for determining patient dose: (a) setting and checking protocol standards and (b) assessing detriment for justification and risk assessment.^1^ It is favorable that the energy imparted is a physical quantity that is not subject to the uncertain estimates of biological effects. The energy imparted value can be used for accurate determination of the effective dose to the patient, especially when specific organ dose values are not of interest.^2^ The energy imparted is also likely to remain the quantity of choice for practical optimization of the image quality and the patient dose^3^, ^4^ as well as the practical quantity of choice for risk estimations.^5^ The Monte Carlo computational method has previously been extensively applied for the estimation of energy imparted to both patient and image receptors^5^ and, in this work, will also be applied in quality control (QC) measurements. The energy imparted is an appropriate choice for QC because it is of major importance for explaining the variations in the dose and the X‐ray energy spectrum.

There is risk associated with high patient doses due to the use of automatic choice of exposure parameters. In radiography, the automatic exposure control (AEC) often adjusts the exposure time to obtain constant detector dose for a given kVp and mA. For fluoroscopy, the system often selects the filtration, kVp and mAs, usually denoted as automatic dose rate control (ADRC). For the sake of simplicity, it is denoted AEC further in this paper, regardless of whether it refers to radiography or fluoroscopy. Application of AEC may result in high milliampere‐seconds (mAs) or suboptimal tube voltage (kV) and filtration. An unintentional increase in the dose may occur due to changes in AEC during equipment servicing or because of dose creep resulting from nonoptimal use of AEC.^6^, ^7^ However, dose increase due to X‐ray system errors that lead to discrepancies between the nominal and true values of the exposure parameters are rare.^8^ The dose to the image detector and the dose‐area product (DAP) are often measured for QC purposes. The DAP is also used in national reference values and must therefore be as accurate as possible. However, due to lack of calibration to the current common beam quality, increased filtration use makes the kerma‐area product (KAP) and the DAP less efficient values for indicating the patient dose.^9^ Standard specifications for medical X‐ray equipment and QC are embodied in various documents from the International Electrotechnical Commission (IEC), Institute of Physics and Engineering in Medicine (IPEM), and American Association of Physicists in Medicine (AAPM). By using the most objective method available, the QC of X‐ray equipment includes every factor that may affect dose and image quality. However, these recommendations do not cover the system performance or energy imparted.

It is crucial to have QC procedures that accurately monitor dose and equipment output. The X‐ray spectrum affects both the imaging properties and the dose. Knowledge of the X‐ray spectra provides important information regarding how the spectra change if the filtration, current or voltage values change. Hence, this knowledge provides an opportunity to calculate the energy imparted, which depends on the X‐ray beam quality, field size, and irradiation geometry.

Models of X‐ray spectra have been available for decades,^10^, ^11^, ^12^ but none have been proposed for use in routine QC.^13^ In 1979, Birch and Marshall computed the bremsstrahlung X‐ray spectra and defined a model that enabled agreement between the computed and measured spectra over the 30‐ to 150‐kV energy range, for target angles between 10° and 30° and a range of filtrations.^14^ More recently, studies on the parameterization of X‐ray absorption,^15^ X‐ray spectra from tungsten anodes,^10^ energy impartments,^3^, ^16^ and electron penetration in X‐ray targets^17^ have been published.

The main aim of the present work was to establish a method for which the calculation of the energy imparted can be used frequently as part of QC for a conventional and fluoroscopic X‐ray laboratory. We expect this method to provide the medical physicist with knowledge of the X‐ray spectra and, thereby, important information about parameters that could affect the dose and image quality.

## 2 THEORY AND METHODS

To derive the energy imparted, an accurate prediction of the photon spectrum emerging from the X‐ray tube is required. In the present work, the theoretical spectra of Birch and Marshall^14^ are combined with data on characteristic radiation from the tungsten target. Together with the attenuation data for Al, Cu, water, and additional materials, these spectra and characteristic radiation data are used to calculate the mean energy, air kerma, energy fluence, photon fluency, and half value layer (HVL). To calculate the energy imparted, a parameterized representation of the air kerma was developed. It is possible to calibrate the actual X‐ray tube by estimating the total filtration (TF) and a calibration factor for the radiation yield, denoted dose factor (DF), for each focal spot by using the least‐square method for the measured air kerma obtained by an X‐ray multifunctional meter (Black Piranha, RTI Electronics, Sweden or similar) against the corresponding calculated values. In this study, Black Piranha was applied. The inaccuracy using Piranha for measurement of kV is ±1.5% in the range 35–160 kV, the inaccuracy for TF is ±10% or ±0.3‐mm Al (1.0‐ to 90‐mm Al), and the inaccuracy for air kerma is ±5% in the range of 1.3 nGy to 650 Gy.

In this study, we present a method for determining the absorbed dose to a homogenous phantom (D_phantom_) using the following four input data: (a) a parameterized model of the X‐ray spectrum, (b) the measurements of the X‐ray tube using a multifunctional X‐ray meter, (c) the exposure parameters, obtained from imaging polymethyl methacrylate (PMMA) slabs of different thicknesses that simulate the patient, using AEC, and (d) the calculation of the dose to water using Monte Carlo simulation for a simple cylindrical geometry and the associated parameterization of the energy fluence. The output is the entrance surface dose (ESD) and the absorbed dose in the phantom (D_phantom_ [µGy]). In addition, a parameterized X‐ray spectrum enables QC of the X‐ray tube by comparing the measurements obtained using a multimeter with the theoretical results for the actual exposure settings and geometry.

### 2.1 Foundation for the method

#### 2.1.1 Energy imparted and air kerma

The energy imparted (*ε*) to the matter in a certain volume is defined by the ICRU^18^ as

(1) $$\varepsilon =R_{\text{in}}‐R_{\text{out}}+\sum Q$$

where *R*_in_ is the radiant energy incident on the volume, namely, the sum of the energies (excluding rest energies) of all the charged and uncharged ionizing particles that enter the volume. *R*_out_ is the radiant energy emerging from the volume, namely, the sum of the energies (excluding rest energies) of all the charged and uncharged ionizing particles that leave the volume. ΣQ is the sum of all the rest mass energy changes for the nuclei and elementary particles in any nuclear transformations that occur in the volume. Considering the X‐ray energies applied in diagnostic imaging, ΣQ is approximately zero, due to the absence of annihilation or pair production, and *R*_in_ is the energy of the primary photon incident on the volume. Therefore, the *ε* to the patient is simplified to^19^

(2) $$\varepsilon ≅R_{\text{in}}\cdot IF$$

where *IF* is the imparted fraction that decreases with increasing energy, which also represents the total energy deposited in a patient undergoing a radiological examination. Assuming that the focal spot can be approximated as a photon point source, the radiant energy that is incident on the area, A, of the registration due to the energy fluence of photons, Ψ, at the angle of incidence, θ to the normal of the area element, *dA*, is described by Ref. [20].

(3) $$R_{\text{in}}=\int_{A}\psi \cos\theta dA=\int_{A}\frac{K_{c,\text{air}}}{{\left(\mu_{\text{en}}/\rho \right)}_{\text{air}}}\cos\theta dA=\frac{\cos\theta }{\mu_{\text{en}}/\rho }\cdot KAP$$

Assuming the X‐rays are perpendicular to the phantom, giving cos θ = 1, the absorbed dose to the homogenous phantom can be written as^18^, ^20^

(4) $$\begin{array}{cc} D_{\text{phantom}} & =\frac{Mean\;energy\;\text{imparted to the phantom}}{\text{mass of the phantom}}\\ & =\frac{K_{A}\cdot \rho /\mu_{\text{en}}\cdot IF}{\text{cm phantom}}\cdot \frac{{\text{SPD}}^{2}}{{\left(SPD+\text{cm phantom}/2\right)}^{2}} \end{array}$$

where *K*_A_ is the air kerma from the primary radiation in the phantom entrance surface, cm phantom is the thickness of the phantom, and SPD is the source‐phantom distance. In this study, the phantom properties for water or PMMA are applied. It is possible to calculate the D_phantom_ by combining the parameterization of the Birch and Marshall spectra, *K_A_*, with the parameterization of the imparted fraction Monte Carlo calculations.^3^, ^5^, ^19^, ^21^

#### 2.1.2 *X*‐*ray spectra*

The X‐ray intensity produced in a solid target is obtained via integration along the electron path to the point where the electron energy, *T*, is equal to the photon energy, *E*, since only electrons with energies greater than *E* can produce a photon of this energy. Birch and Marshall's calculation of the bremsstrahlung X‐ray spectra gives the energy intensity, *I*(*E*), at the photon energy, *E*, generated by an electron traveling a distance, *dx*, within an element as^14^

(5) $$I\left(E\right)=\frac{\rho N}{A}\int_{T_{0}}^{E}\left(1+\frac{T}{m_{0}c^{2}}\right)Q{\left(\frac{\mathrm{dT}}{\mathrm{dx}}\right)}^{‐1}\cdot \exp\left(\frac{‐\mu (E)}{\rho C}\left(T_{0}^{2}‐T^{2}\right)\cot\theta \right)dt$$

for a tungsten target with the attenuation coefficient *µ*(*E*). The term [1 + (T/m_0_c^2^)] corrects for relativistic effects, *θ* is the target angle, *N* is Avogadro's number, *ρ* is the density, *A* is the atomic weight, and *Q* is the X‐ray energy intensity per unit energy interval per incident electron flux per atom. *dT*/*dx* is given by the Bethe equation,^22^ and *C* is the Thomson–Whiddington constant.^14^

The Birch and Marshall's theoretical spectra, supplemented with data for the characteristic radiation from the tungsten target, were integrated into a Microsoft Office Excel 2010 spreadsheet (SPECTRUM) for general and daily use. The input parameters were the kV, mAs, distance between the X‐ray tube and the detector, referred to herein as the source‐to‐image distances (SIDs), and thickness of the filtration or object in the X‐ray beam. The spectra may be computed for any desired target angle (1–25°) and tube voltage between 40 and 150 kV with the use of attenuation data for these materials: Al, Cu, Pb, Be, C, Sn, I, water, PMMA, air, muscle, and bone. This spreadsheet provides the mean photon energy, air kerma, energy fluency, photon fluency, and HVL for an X‐ray beam traveling through a given combination of these materials.

#### 2.1.3 Monte Carlo simulations

**FIGURE 1:**
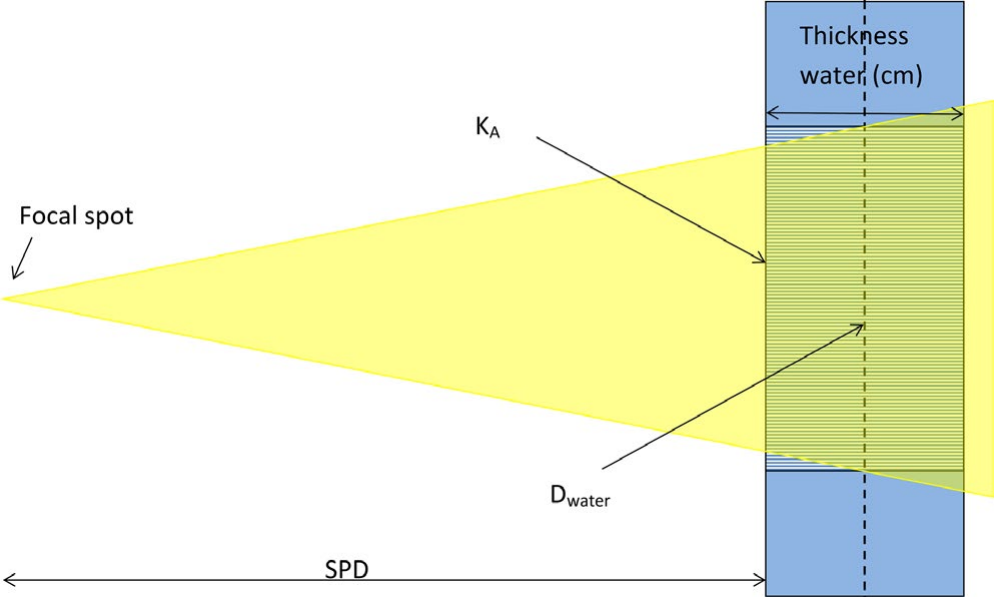
Geometry for calculating the dose to a homogenous phantom. A cone shaped X‐ray beam origin from the focal spot enters the phantom with the central beam coincide with the cylinder axis of the water phantom. The beam base is circular with a diameter smaller than the water phantom. The dose calculation is based on the volume of water depicted by the hatched area. The D_phantom_ is defined as the energy imparted divided by the mass of a water cylinder with height equal to the phantom thickness used at QC and a surface area equal to the area of the X‐ray beam in the center of the phantom indicated by the broken line

The Monte Carlo calculations were designed specifically for these spectra and were performed using cylindrical symmetry, to decrease the computational time. In the simulations, a cylindrical water phantom was centered inside a larger cylindrical air phantom. A cone‐shaped X‐ray beam originating from the focal spot entered the phantom in such a manner that the central radiation of the beam coincided with the cylinder axes. Both phantoms were divided into ring‐shaped dose‐accumulation voxels. The air phantom was 120 voxels high with a 60 voxel radius, and the width of a voxel was set to 20 mm. The height of the water phantom varied between 40 and 300 mm, and the radius was set to 120 mm. The SPD for the water phantom was set to 1500 mm. The field radius of the X‐ray beam entering the water phantom was 50 mm; see Figure 1.

The incident X‐ray photons had energies ranging from 1 to 150 keV at increments of 1 keV. All the interactions, such as Compton scattering, coherent, and photoelectrical effects, were tracked through the air and water, and all the energy losses were registered until the photons came to rest or left the air phantom. The atomic interaction cross sections and form factors were from Hubbell et al.^23^ and Johns and Cunningham,^24^ and 10^9^ photons were simulated in total. The energy loss from primary and scattered photons was recorded separately. The energy loss from primary photons was recorded in special voxels that were adapted to the cone‐shape of the X‐ray beam to avoid unnecessary partial volume effects and to reduce the amount of stored data.

### 2.2 Parameterization

To achieve an applicable theoretical calculation of air kerma and absorbed dose to a homogenous phantom for QC purposes, they were parameterized as function of standard exposure parameters applied in an X‐ray laboratory.

#### 2.2.1 Parameterization of air kerma

In the proposed method, the calculation of the X‐ray spectrum emerging from an X‐ray tube was obtained by parameterization of the air kerma, *K_A_*. This was done in two steps. Step one is a parameterization of a preliminary air kerma, K_A,prelim_, where Cu filtration is excluded. This parameterization was given by

(6) $$K_{A,pre\lim}=\sum_{i=0}^{4}\sum_{j=0}^{4}a_{\text{ij}}U^{i}{\left(\text{TF}\right)}^{‐j}$$

where *U* is the tube voltage and *TF* is the total filtration without added Cu filters (mm Al). This calculated *TF* includes filtration through the X‐ray tube glass and Al‐filters, if built‐in or added. The 25 *a_ij_* values were obtained from the best fit of a rational function with fourth‐order polynomials for any voltage and *TF* values.

Step two is a parameterization of the transmission through Cu filters (mm Cu) with *K_A_*_,_ *_prelim_* as a reference and is given by

(7) $$T_{\mathrm{Cu}}=\exp\left(\sum_{i=0}^{3}\sum_{j=0}^{2}\sum_{k=1}^{4}a_{\mathrm{ijk}}U^{i}{\left(\mathrm{TF}\right)}^{j}{\left(\mathrm{Cu}\right)}^{k}\right)$$

The 48 *a_ijk_* values were obtained from the best fit of a three‐dimensional exponential polynomial for any voltage, *TF* (without Cu) and Cu filtration values.

Applying the input parameter tube voltage (kV), mAs, and the distance between the focal spot of the X‐ray tube and the detector (SDD), K_A_ is given by

(8) $$K_{A}=\frac{mAs\cdot DF\cdot T_{\mathrm{Cu}}\cdot K_{A,pre\lim}}{SDD^{2}},$$

where DF is a calibration factor of the radiation yield given for the focal spot in a specific X‐ray tube. The parameterized SPECTRUM was implemented in an Excel sheet for QC of the X‐ray tube. Using the developed method, for each X‐ray tube, the parameterized SPECTRUM is calibrated by calculating the DF for each focal spot and the TF of the system. The calibration is conducted by finding the TF and DF that minimize the sum of the weighted squared logarithmic deviations between the measured and calculated *K_A_*. In the Excel sheet, TF is the filtration through the X‐ray tube glass and built‐in filtration, and added Al is recorded separately. However, in the estimation of K_A,prelim_ according to Equation (6), the sum of TF and the external added Al is applied. The nominal input values includes the distance from the source to the X‐ray meter, referred to herein as the source‐to‐detector distance (SDD), additional mm filtration of Al or Cu, applied focal spot (small, large, or fluoroscopic), kV, mA, and ms or mAs and pulses per second (pps) for fluoroscopic imaging. Input values from the X‐ray multimeter measurements are tube voltage in kV, exposure time in ms and air kerma in µGy for radiography and µGy/s for fluoroscopy. The outputs include the theoretical air kerma at an SDD of 1 m, the measured value for the air kerma at 1 m, the associated deviation, the discrepancies between the nominal and measured values for the kV and exposure time, and the DF and TF. If any earlier estimates for TF and DF exist, they are included in the spreadsheet. If it is the very first measurements for the actual X‐ray tube a measured TF can be input as the first guess for TF in the model, and the DF values are set to one. Previous measurements of the X‐ray tube can be included in the spreadsheet; however, they can be emphasized less. In the present work, the weighting was performed by reducing the influence of the deviation between the measured and calculated air kerma on the determination of TF and DF. The DF and TF values are applied in an Excel sheet to determine the dose to the homogenous phantom based on recorded exposure parameters, which were applied using AEC as described in Section 2.2.3.

#### 2.2.2 Parameterization of energy fluence

As shown in previously published studies, the fluence can be parameterized.^10^, ^15^, ^17^ In our study, the parameterization of the energy fluence was applied for the Monte Carlo calculations and given as

(9) $$\Psi \left(U,E\right)=\sum_{i=0}^{3}\sum_{j=1}^{4}a_{\text{ij}}U^{i}{\left(1‐\frac{E}{U}\right)}^{j}$$

where *U* is the notation for tube voltage (kV) and *E* is photon energy (keV). The values of *a_ij_* were derived from the best fit of a two‐dimensional polynomial for any values of the voltage and photon energy. The simulated air kerma from the primary photons multiplied by the beam cone‐shaped area yielded the KAP.

#### 2.2.3 Parameterization of the imparted fraction in the water phantom

The imparted fraction of the energy was summed over the total water phantom given by the Monte Carlo calculations. The primary photons that register as escaped or unhindered in the Monte Carlo data were excluded since they do not contribute to the energy. The imparted fraction was calculated using Equation (10)19:

(10) $$\text{IF}=\frac{\varepsilon }{R_{\text{in}}}={\text{IF}}_{s}+{\text{IF}}_{p}=\left(1‐T_{p}\right)\cdot S_{\varepsilon }+\left(1‐T_{p}\right)\cdot P_{\varepsilon }$$

where *T_p_* is the transmitted energy fraction of the primary photons and Sε and Pε are the fractions of the ε from the scattered and primary photons, respectively. On this basis, the parameterization of the transmitted energy from the primary photons, Tp, in the water phantom was given by

(11) $$T_{P}=e^{‐\sum_{i=‐2}^{2}\sum_{j=‐2}^{2}a_{\mathrm{ij}}{\left(\frac{\Psi }{K_{A}}\right)}^{i}t^{\left(1+\frac{j}{10}\right)}}$$

where *t* is the thickness of the water phantom, Ψ is the energy fluence from the Monte Carlo calculations and *K_A_* is the air kerma derived from the Birch and Marshall spectra. The *a_ij_* values were obtained from the best fit of a three‐dimensional exponential polynomial for any values of the energy fluence, air kerma, and thickness of a water phantom.

The parameterization of the *ε* from both the primary photons and the scattered photons was based on the same equation:

(12) $$P_{\varepsilon }=S_{\varepsilon }=\sum_{i=0}^{3}\sum_{j=0}^{5}a_{\text{ij}}\left(U^{i}+{\left(\frac{t}{t+U/10}\right)}^{i+1}\right){\left(\frac{\Psi }{K_{A}}\right)}^{j}$$

where *t* is the thickness of the water phantom, U is the tube voltage, Ψ is the energy fluence, and *K_A_* is the air kerma. The a_ij_ values were obtained from the best fit of a three‐dimensional exponential polynomial for any values of energy fluence, air kerma, and thickness of a water phantom. However, the a_ij_ values were different for Pε and Sε. The corresponding values for the air kerma and energy fluence were calculated for different beam qualities. This relationship was parameterized as

(13) $$\Psi /K_{A}=\sum_{i=0}^{3}\sum_{j=0}^{3}a_{\text{ij}}U^{i}{\left(\text{TF}\right)}^{j}$$

and

(14) $$\text{TF}={\text{TF}}_{\text{Al}}+{\text{TF}}_{\text{Cu}}\left[\text{mmAl}\right]$$

where

(15) $${\text{TF}}_{\text{Cu}}\left[\text{mmAl}\right]=\text{Cu}\sum_{i=0}^{3}a_{i}U^{i}$$

where Cu is the thickness of the Cu filtration, given in mm, and TF is the total filtration through the X‐ray glass and built‐in or added filtration of both Al and Cu.

Using Eq. (10), the dose to the homogenous phantom was determined by imaging PMMA slabs of different thicknesses using AEC. When applying the developed method made for purpose of QC of AEC, 12‐, 16‐, 20‐, and 24‐cm PMMA slabs are imaged using the typical clinical protocols for each laboratory. These PMMA thicknesses are chosen to represent a child of 30 kg and 138 cm, a youth of 45 kg and 160 cm, a standard adult of 60 kg and 170 cm, and a large adult of 80 kg and 170 cm, respectively. The input parameters are TF (through X‐ray glass and built‐in filtration) and DF, as described in Section 2.2.1, and the additional filtration (mm Al/Cu), focal spot, kV, mAs (for fluoroscopy pps, pulse width, mA per pulse), thickness of PMMA, SID, and SPD values must be recorded at the time of the examination.

### 2.3 Validation of the method

In order to validate the method, the SPECTRUM, the parameterized air kerma, and the dose to homogenous phantom were compared to previously published methods or measurements.

The proposed method to calculate air kerma was verified by comparing SPECTRUM to two commercial spectra, SpekCalc^12^ and IPEM report 79,^11^ and Black Piranha measurements (RTI Electronics, Sweden) using the associated software Ocean Professional (Version 2018.02.06.264, RTI Electronic, Sweden). The deviation between the measured air kerma and the theoretical air kerma was compared for the parameterized model, IPEM report 79 and SpekCalc.

The absorbed dose to a homogenous phantom was verified through calculations using PCXMC.^25^ PCXMC was used to calculate the absorbed energy fraction of those simulated patients. The parameters used for these calculations were obtained from the exposure parameters recorded using AEC (see Table 1) and the TF harvested from the characterization of the X‐ray tube (see Section 2.2). Additionally, the actual anode angle was an input (Table 2). The simulations in PCXMC include 50 000 photons per energy level.

**TABLE 1:** The choice of exposure parameters using AEC (additional filtration in mm Al and mm Cu, kV, mAs) for four different systems imaging 12‐, 16‐, 20‐, and 24‐cm PMMA at the given source–phantom distance (SPD) and source–image distance (SID)

Focus	DF	TF	Al (mm)	Cu (mm)	kV	mAs	PMMA (cm)	SPD (cm)	SID (cm)	D_phantom_ (µGy)	Absorbed energy fraction (IF)
Multifunctional system: Siemens Luminos dRF Max fluorospot compact table, year of installation: 2014 (Lab 1a)											
SF	0.98	3.25			109	1.5	12	129.5	150	62	0.49
SF	0.98	3.25			109	3.4	16	125.5	150	124	0.56
SF	0.98	3.25			109	7.7	20	121.5	150	249	0.60
SF	0.98	3.25			109	17.8	24	117.5	150	516	0.62
Multifunctional system: Siemens Luminos dRF Max wall stand, year of installation: 2014 (Lab 1b)											
SF	1.02	3.11			121	2.1	12	164.5	180	74	0.47
SF	1.02	3.11			121	5.1	16	160.5	180	157	0.53
SF	1.02	3.11			121	11.2	20	156.5	180	305	0.58
SF	1.02	3.11			121	24.7	24	152.5	180	599	0.60
Conventional X‐ray system: Arcoma Triathlon T3 precision, year of installation: 2019 (Lab 2)											
SF	1.08	3.30			125	3	12	166.0	180	130	0.46
SF	1.08	3.30			125	8	16	162.0	180	255	0.53
SF	1.08	3.30			125	17	20	158.0	180	503	0.57
SF	1.08	3.30			125	38	24	154.0	180	999	0.60
Interventional system: Philips Allura Clarity FD 20, year of installation: 2015 (Lab 3)											
fl	1.13	3.40	1	0.9	71	1.8	12	83.0	120	3	0.51
SF	1.12	3.40	1	0.4	80	6.0	12	83.0	120	64	0.50
Fl	1.12	3.40	1	0.9	77	2.5	16	83.0	120	6	0.54
SF	1.12	3.40	1	0.4	80	12.0	16	83.0	120	101	0.56
fl	1.12	3.40	1	0.9	84	3.0	20	83.0	120	11	0.54
SF	1.12	3.40	1	0.4	80	28.0	20	83.0	120	190	0.59
fl	1.12	3.40	1	0.9	95	3.0	24	83.0	120	17	0.54
SF	1.12	3.40	1	0.4	80	62.0	24	83.0	120	344	0.60
C‐arm: Ziehm Vision FD Vario 3D, year of installation: 2018 (Lab 4)											
fl	1.17	4.51		0.1	65	0.9	12	90.9	107	11	0.61
fl	1.17	4.51		0.1	65	2.0	16	86.9	107	21	0.67
fl	1.17	4.51		0.1	65	4.6	20	82.9	107	42	0.70
fl	1.17	4.51		0.1	70	6.5	24	78.9	107	70	0.69

Note

The applied focus (SF = small focus, fl = fluoroscopic focus), the associated dose factor (DF) and the estimated total filtration through X‐ray tube glass and built in filtration (TF) are given in the first three columns. A few results are also included, such as the calculated dose to homogenous phantom (D_phantom_) and the corresponding absorbed energy fraction.

**TABLE 2:** The main technical characteristics of the generator and X‐ray tube for each system nominal focal spot size is large (LF), small (SF) or fluoroscopic focal spot (fl). In addition, the deviation from the linearity of the measured µGy/mAs versus kV^2^ for each focus is included

Imaging system	X‐ray generator	X‐ray tube	Anode angle	Nominal focal spot size (mm)	Deviation from linearity (1‐*R* ^2^)
Siemens Luminos dRF Max fluorospot compact table	Polydoros F 80 (150 kV/80 kW)	OPTITOP 150/40/80HC−100	12°	SF 0.6	4.1%
				LF 1.0	3.0%
				fl	24.3%
Siemens Luminos dRF Max wall stand	Polydoros F 80 (150 kV/80 kW)	OPTITOP 150/40/80HC−100	12°	SF 0.6	0.6%
				LF 1.0	0.5%
Arcoma Triathlon T3 Precision	CPI 65 kW DAP, CMP 200	Varian A−292 Housing tupe B−130	12°	SF 0.6	0.5%
				LF 1.2	0.4%
Philips Allura Clarity FD 20	Certeray iX	MRC 200 0407 ROT‐GS 1004	11°	SF 0.4	0.3%
				LF 0.7	0.3%
Ziehm Vision FD Vario 3D	Ziehm Imaging, Vision Pulse	Varian RAD−15,	10°	SF 0.3	1.6%*
				LF 0.6	

Note

The * indicates cases where the used focal spot is not known.

This paper includes results from four different laboratories representing five different X‐ray tubes and generators, a variety of imaging technique parameter settings (kVp, filtration, mA, exposure time, exposure rate and pulses), and vendors, as described in Tables 1 and 2. The data presented for each laboratory were acquired on one session within a couple of hours, except for laboratory one and four where three and two sessions are included, respectively.

## 3 RESULTS

In this study, the parametrization of the air kerma is embedded in an Excel spreadsheet. The spreadsheet provides immediate feedback on the multimeter measurements of the X‐ray tube compared to the theory. The measurement conditions, including the SDD, kV, mAs (or mA and ms), pulses for fluoroscopic imaging and filtration, are recorded in the spreadsheet. This method is the most convenient if the results from the multimeter are automatically transferred to the spreadsheet. The input information to determine the absorbed dose to the homogenous phantom is acquired by imaging a phantom of different thicknesses using AEC. Hence, one can perform QC of the X‐ray tube and obtain easy access to the energy imparted to a homogenous phantom, which is shown to correspond to actual patient doses.

### 3.1 Spectrum

**FIGURE 2:**
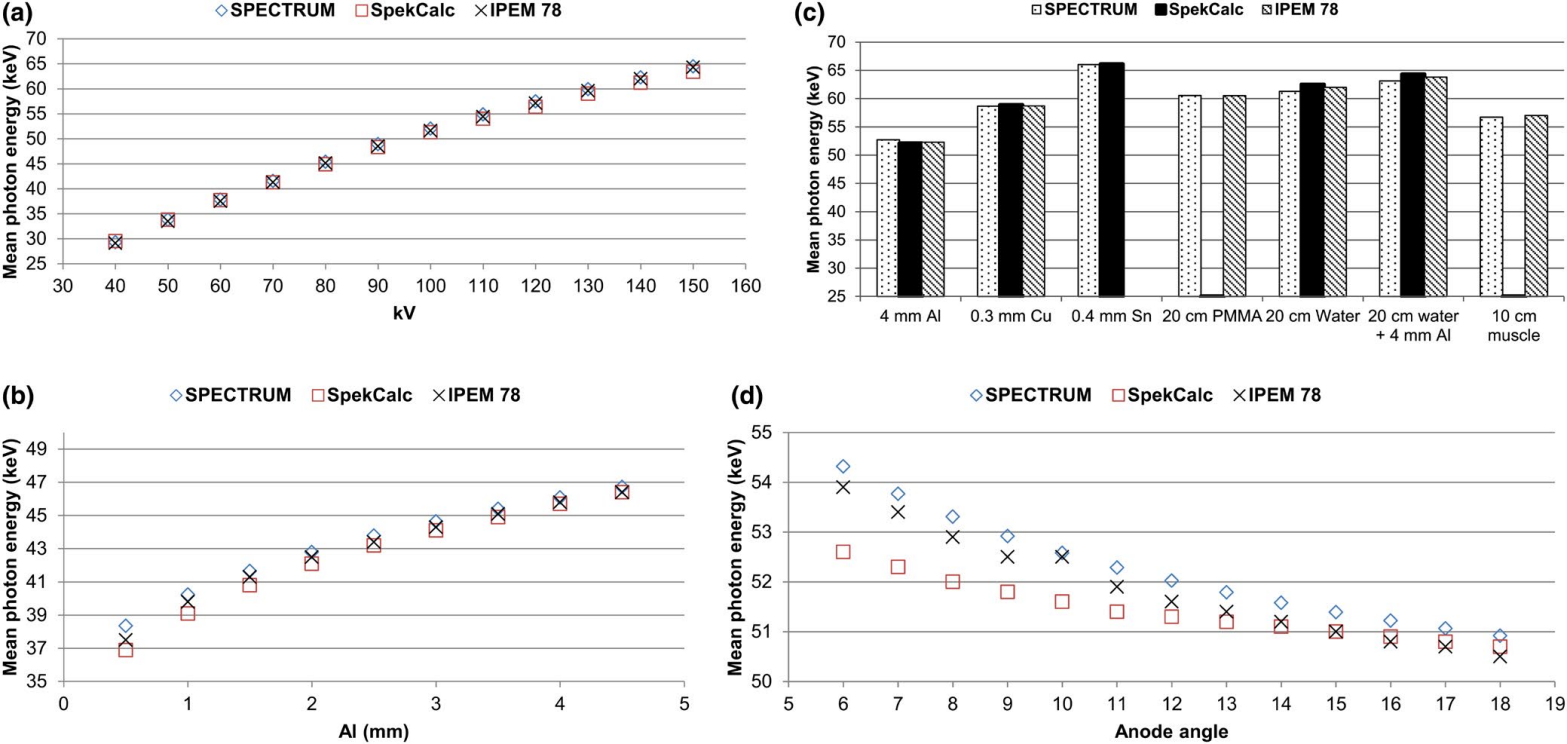
(a) The mean photon energy versus tube voltage using an anode target at 12° and a 3.5‐mm total Al filtration. (b) The mean photon energy versus total Al filtration using an anode target at 12° and 80 kV. (c) The mean photon energy versus different materials and thicknesses using anode target 12° and 100 kV. PMMA and muscle are not available in SpekCalc, and Sn is not included in IPEM 78. (d) The mean photon energy versus anode angle using 100 kV and a 3.5‐mm total Al filtration

A comparison between the spectrum calculated using the SPECTRUM Excel sheet and the two commercially available X‐ray spectra, IPEM 78 (version Spectrum Processor 3.0, August 2015)^11^ and SpekCalc,^12^ is shown in Figure 2. This figure shows the mean photon energy obtained using the following settings: (a) different kV at a 3.5‐mm Al filtration, (b) 80 kV for different thicknesses of the Al filtration, (c) different materials at 100 kV, and (d) different anode angles at 100 kV. Compared to SpekCalc, the absolute percentage differences vary from 0.05% to 3.9% with an average of 1.4% (Figure 2a–d). The deviation from IPEM report 78 varies from 0.02% to 2.3% with an average of 0.74% (Figure 2a–d). The spectra were parameterized for calculation of the imparted fraction for target angles of 10°, 12°, and 15°, kV (50–150 kV) with Al (2.2–8 mm), Cu (0–1 mm), PMMA, and water materials.

### 3.2 QC and characterization of the X‐ray tube

**FIGURE 3:**
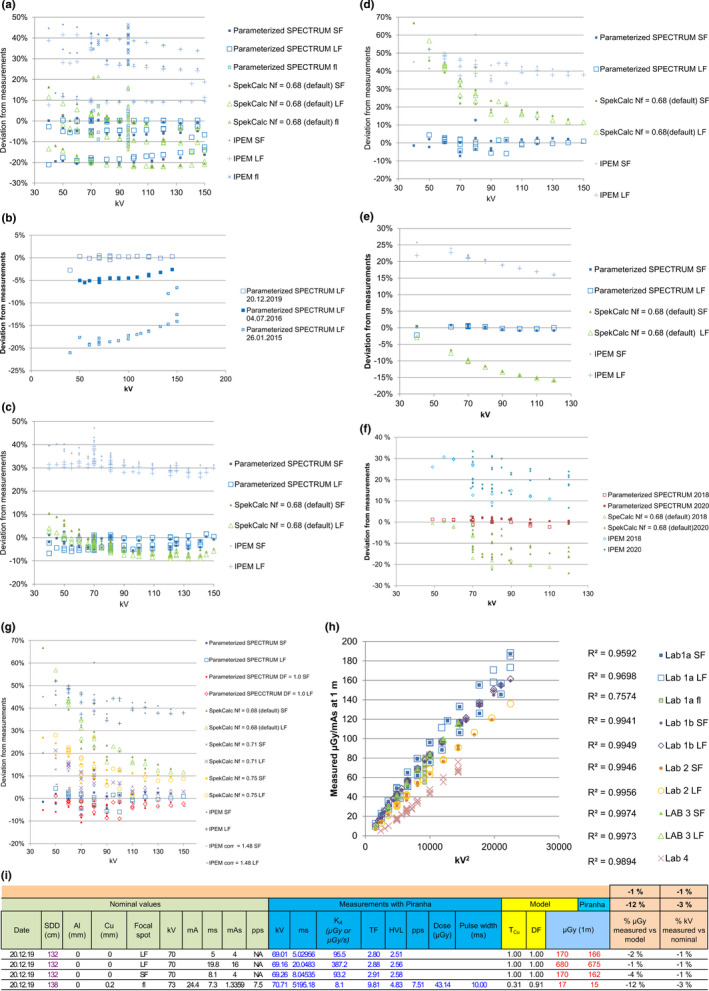
(a–g) The deviation between the theoretical and the measured air kerma versus applied kV for the three different spectra models: parameterized SPECTRUM, SpekCalc and IPEM 78 for the four systems. Each dot is one exposure. (a) The multifunctional table (Lab 1a), (b) an excerpt of data from a) versus date of measurement, (c) wallstand (Lab 1b), (d) a conventional (Lab 2), (e) an interventional (Lab 3), and (f) a C‐arm (Lab 4). (g) The deviation between the measured air kerma versus kV for different DF for the parameterized SPECTRUM and Nf for SpekCalc, and a corrected IPEM. (h) The measured radiation output versus kV^2^ for the X‐ray systems included in this study, and the associated coefficient of determination (*R* ^2^). (i) The layout of the spreadsheet for the measurements conducted with a multimeter to calibrate the X‐ray tube by determining the dose factor (DF) for each focal spot and the total filtration (TF) of the system. The nominal values should be entered in the green columns and the data in the dark blue columns should be automatically populated from the X‐ray multimeter. The spreadsheet then compares the measured air kerma and voltage with the modeled air kerma and the nominal values for kV. For fluoroscopic imaging *K*_A_ (from the mulitmeter) is the air kerma doserate and for radiography it is the total air kerma from one exposure

The DF and TF for four different systems are shown in Table 1, and Figure 3a–g shows the discrepancies between the theoretical and measured air kerma versus kV for the three models (parameterized SPECTRUM, SpekCalc, and IPEM report 78) and the five different X‐ray systems (described in Tables 1 and 2). The parameterized SPECTRUM agrees with the measured values, for all the measurements except the Lab 1a measurements, within 10% (Figure 3a,b). The deviation from measurements using a large focus (LF) represents three different levels, which correspond to three sessions on different measurement days (Figure 3b). The largest deviation occurs for measurements from January 2015, followed by measurements from July 2016, and the best fit is for the data from December 2019. In addition, Figure 3g illustrates the models with different DFs for the X‐ray system given in Figure 3d. The results for DF = 1 and DF = 1.038, which represent the optimized DF from the model, are shown for the parameterized SPECTRUM. SpekCalc uses a parameter, Nf, that represents an output normalization factor. Nf can be adjusted to match the predicted output to the measured output for a particular tube. It is expected that Nf is approximately 1 and Nf = 0.68 is a default value describing one particular tube over a range of tube potentials. Nf = 0.68, 0.71, and 0.75 were chosen to minimize the deviation between the measured and calculated radiation outputs. Because IPEM 78 does not include a DF, over the range of the tube potentials, the mean value of the radiation output from IPEM 78 divided by the output from the parameterized SPECTRUM was applied as a correction factor, which gives IPEM corr in Figure 3g. As the DF changes, the discrepancy moves up or down along the *y*‐axis. Figure 3h presents the variation in the radiation output for each X‐ray tube. It is an adequate approximation to consider the radiation output to be proportional to the square of the tube voltage.^26^ Therefore, the regression value, 1‐*R* ^2^ (see Table 2), accounts for the variance that is not explained by the nominal kV and indicates inherent variation in the system. As the 1‐*R* ^2^ value increases, a higher deviation from the measured values is expected. Figure 3i presents the layout of the spreadsheet including the parameterization of the transmission through added Cu filters (Equation 7) and the air kerma (Equation 8). The spreadsheets calculate TF and DF by minimizing the deviation between the measured and calculated air kerma. For situations in which repeated measurements are performed on the same equipment, the spreadsheets contain earlier estimates of the TF and DF. The estimation of TF and DF requires approximately 20–30 measurements in the available kV and filtration range.

### 3.3 Determination of dose to the homogenous phantom, *D*_phantom_

**FIGURE 4:**
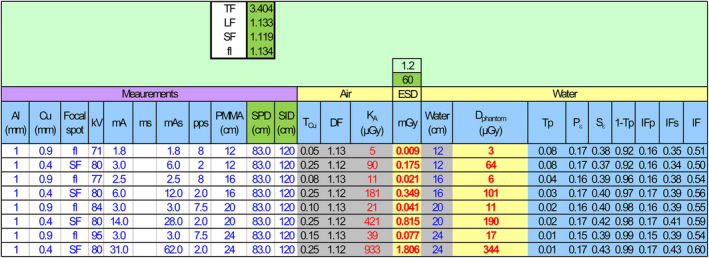
Layout for the spreadsheet used to determine the dose to the homogenous phantom

The spreadsheet for determining the dose to the homogenous phantom is shown in Figure 4. External added Al filtration is recorded in the first column, and the TF value given in the matrix (tenth column) includes only filtration through the X‐ray tube glass and Al‐filters, if built‐in. Some typical results are shown above in Table 1 for PMMA thicknesses of 12, 16, 20, and 24 cm and four different X‐ray systems. The doses are typically between 60 and 1000 µGy for exposure imaging and less than 100 µGy/s for fluoroscopic imaging.

### 3.4 Comparison of the *D*_phantom_ and dose calculated by PCXMC

**FIGURE 5:**
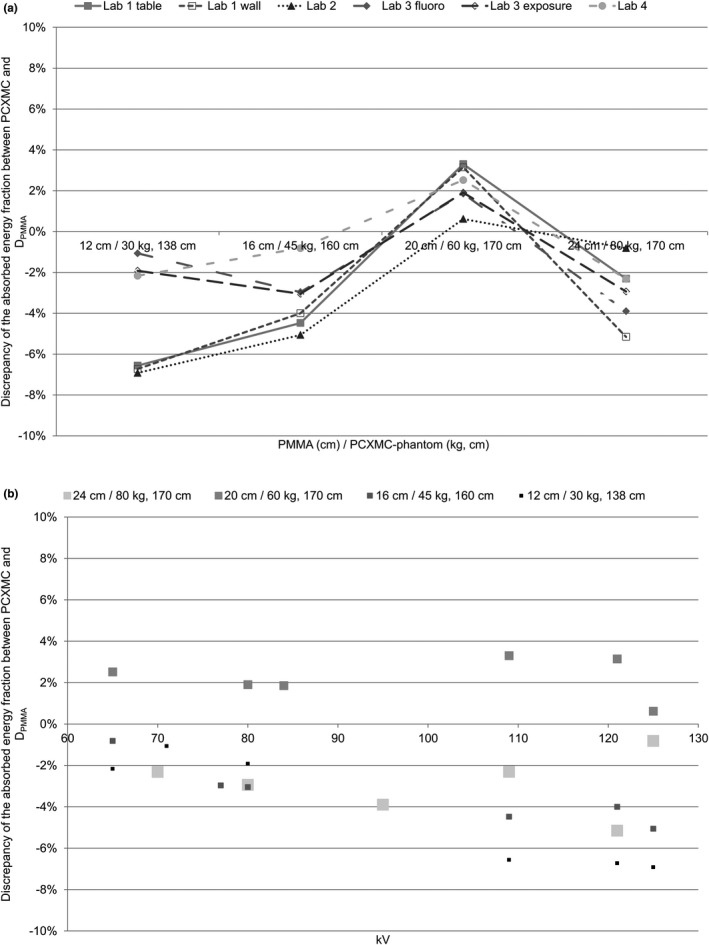
The discrepancy results, plotted as deviations of the energy fraction between PCXMC and D_phantom_ for four different X‐ray systems, are represented (a) versus the thickness of the PMMA/phantom size and (b) versus the kV for the various combinations of the PMMA and phantom size

The discrepancy between the absorbed energy fraction for PCXMC and D_phantom_ is shown in Figure 5. In all laboratories, the discrepancy is 7% or less for the kV and PMMA thicknesses/PCXMC phantom. Figure 5b shows that the discrepancy increases with increasing kV and with decreasing thickness.

## 4 DISCUSSION

In this study, using standard patient protocols with AEC in an X‐ray examination room, we present a unique and practical method for performing QC of X‐ray tubes and determining the absorbed doses to a homogenous phantom. Knowledge of the X‐ray spectra provides important information about how the spectra will shift with changes in filtration, current or voltage. By establishing a QC method for calculating the *ε* based on the equipment‐specific X‐ray spectrum, valuable information on the conditions that can affect the dose for a specific lab becomes easily accessible. This information is also useful for the optimization of X‐ray systems, particularly when a new system is installed. The method is embedded into an Excel spreadsheet, which allows the user to perform measurements using a multifunctional X‐ray meter to characterize the spectrum and calibrate for a specific X‐ray tube. Depending on user preference, the multimeter results can be automatically transferred to the spreadsheet. Imaging of PMMA using AEC can be performed and the system's choice of exposure parameters, such as the kV, mAs, filtration, SID, and SPD, can also be recorded in the spreadsheet. In this way, both the absorbed dose to the homogeneous phantom and the entrance dose are calculated directly.

The mean energy for the spectrum derived in the present study was compared to the previously published spectra, IPEM report 78^11^ and SpekCalc.^12^ Considering the kV, filtration (mm Al) and material, Figure 2 shows excellent agreement between the mean photon energies. The discrepancy between SPECTRUM and SpekCalc is approximately the same as the discrepancy reported between SpekCalc and IPEM 78.^11^, ^27^ SPECTRUM is more similar to IPEM 78, as they both have a harder spectrum, with a higher mean energy, than SpekCalc. SpekCalc is based on Monte Carlo simulations,^28^ whereas IPEM report 78 and SPECTRUM are based on Equation (5).

This work introduces an original method for interpreting the measurements performed during QC testing. Besides that the X‐ray spectra can be easily calculated, the greatest benefit of this method is that the air kerma is calculated when the nominal kV, mAs, and filtrations are provided. The user obtains an immediate comparison between the measured and calculated air kerma and kilovoltage. Figure 3a–f shows that the parameterized SPECTRUM matches the measured air kerma reasonably well, within 10%, for all systems, and the parameterized SPECTRUM is closer to the measured value than IPEM 78 and SpekCalc. One exception is the Lab 1 data (Figure 3a,b), where there are larger deviations for the measurements performed in 2015 and 2016 than for the measurements from 2019. In the parameterized SPECTRUM, newer measurements are weighted more than older measurements. This is motivated by the fact that the radiation output varies due to factors such as power supply characteristics and inherent filtration,^29^ which may change over time. The authors have also noticed that the radiation output is dependent on the focal spot and calibration of the generator. Hence, the radiation output may change after system servicing. Figure 3g shows that, by adjusting Nf, the SpekCalc result comes closer to the measured value, but its deviations seems to be dependent on kV. Due to the assumption that electrons instantaneously attain a diffuse directional distribution, the model employed in SpekCalc overestimates the bremsstrahlung contribution to the air kerma by 10%–30%, with even higher deviation for low incident electron energies.^30^ The deviation for IPEM 78 has a flatter curve, similar to the parameterized SPECTRUM. However, the IPEM 78 calculator does not include a DF for adjusting the measured output. Despite this disadvantage, the IPEM 78 spectrum can be adjusted by the mean value of its radiation output divided by the output from the parameterized SPECTRUM over the range of tube potentials, which is 1.38 for the lab in Figure 3g. The resulting IPEM corr is also close to the measured values.

The parameterized SPECTRUM, IPEM 78, and SpekCalc always calculate the same value for the same input. However, the output from an X‐ray tube may vary for equal nominal exposure values. This is illustrated in Figure 3h. Most often, the variation from different X‐ray systems is less than 5%. A 5% variation in the radiation output can be associated with an approximately 2%–3% deviation between the nominal and measured kV. This range is considered a typical deviation between the nominal and measured kV. Some older mobile X‐ray systems and some fluoroscopic X‐ray tubes may have larger deviations of 5%–10% from the nominal kV. This deviation corresponds to a reduction in the air kerma of approximately 10%–20%. If the applied focal spot is unknown and the radiation output depends on the focal spot, then the inherent variation will increase and might be as much as 20%. The parameterized SPECTRUM deviates less than 10% from the measured air kerma. Hence, the presented method is well suited for X‐ray tube QC purposes.

An X‐ray multimeter made up of semi conductive material might have an energy‐dependent response. However, most modern X‐ray multimeters automatically correct for this dependency and the accuracy of kV and air kerma is typically within 5%. An error of −5% in kV results in ~6% decrease in estimated TF. D_phantom_ is then increased by ~4% on average for the tube voltage range 50–130 kV. However, the DF increases ~5% for a 5% reduction in kV. When the kV erroneously increases 5% the DF is reduced by more than 10% and is out of range, which indicates that something is wrong. However, the range of airkerma is about the same, but it is more asymmetrical. The D_phantom_ is proportional to DF and will change according to changes in DF. A variation of ±10% of TF gives on average ±6% of D_phantom_ in the tube voltage range 50–130 kV.

AAPM Task group 151^13^ discusses methods to assess patient doses in digital radiography. These methods involve calculating the effective dose to any phantom size using known technical factors from manual exposure, but this publication further states that the techniques are not practical due to their complexity. The task group also considers parameters to perform exposure analysis, such as the exposure index, ESD and DAP. The DAP is the desirable quantity because it accounts for all the factors that influence the amount of radiation striking a patient, the output from the X‐ray tube and the size of the X‐ray field. Although the DAP is frequently used to generate national reference levels, all the conversion factors, from the DAP to the ε, suffer from dependence on the beam quality.^2^, ^31^, ^32^ In addition, the specific combination of kV and filter thicknesses in the X‐ray system might be considerably different from the combinations utilized by radiation dosimeter calibration laboratories.^9^ As given in the IAEA handbook, the intrinsic error for a typical DAP meter for fluoroscopy and radiography is 10%. In addition, there might be 8% variation due to variation of energy response.^33^ Hence, the suspension level for DAP is a deviation of more than 35% between measured and indicated DAP.^34^

Figure 5 shows that there is good agreement between the absorbed energy fractions calculated using the present method and the PCXMC. Except for the smallest phantom (12‐cm PMMA) and highest kV, where the presented method calculated lower doses, the deviation is less than 5%. This behavior might be explained by the fact that the PCXMC overestimates the doses for pediatric patients.^35^ The effective doses for the phantom study are comparative values and not absolute values, and the discrepancy between *D*_phantom_ and the dose from the PCXMC change as the chosen phantom weight (kg) and length (cm) change. Because all types of X‐ray laboratories can be controlled by the same method, *D*_phantom_ can also be used to compare examinations, or protocols, at different laboratories. The doubling of *D*_phantom_ indicates twice the absorbed dose to the patient, regardless of the applied exposure parameters. The reason for this large dose effect is that these measurements reveal the differences between the different laboratories and different modalities regarding the applied kV, mAs, and filtrations using AEC. The presented method is well suited to estimate the energy absorbed by a homogeneous phantom, *D*_phantom_, which is useful for application in acceptance tests and QCs of AEC systems. Typically, there is no need to perform QC measurements of the TF and reproducibility of the radiation output using exactly the same geometry and exposure settings. This is because the TF and reproducibility of the radiation output are obtained by the same measurements that are performed to estimate the TF and DF for determination of the dose to a homogenous phantom. Another advantage of this method is that there is no need for a multimeter in the image field when AEC is assessed.

The Birch and Marshall theoretical spectra^14^ were the foundation of this method, along with the Monte Carlo calculations, and the parameterization of those. The parameterization enabled direct feedback on the measurement of an X‐ray tube using a multimeter. With this method, it is therefore easier to reveal erroneous measurements due to errors in the system or due to wrong assumptions about the exposure setting, geometry, or protocols. Even though there is not a risk for deterministic skin effects in conventional radiography, it is quite common to optimize conventional X‐ray imaging by using the ESD (air kerma) as a risk factor. However, the calculation of the absorbed dose to a phantom or effective dose is a more appropriate indicator of risk. Using the presented method, it is easy to avoid the pitfall that a reduced skin dose does not always equate to a reduced absorbed dose to the patient. For routine QC, D_phantom_ is calculated for representative procedures to notice changes from one control to the next. If the X‐ray equipment or the protocol changes, it may affect the D_phantom_ and image quality. This method is an effective tool to optimize protocols and compare protocols within and between X‐ray systems, especially for new systems lacking exposure parameters and dose values from patient examinations. For instance, if an X‐ray system is replaced by a new system from a vendor that promises to give a lower dose by a certain percent, this dose can be verified using the proposed method to determine the actual dose to the homogenous phantom. Hence, protocols can be adjusted with respect to dose before the first patient is even imaged. However, the concept of a dose to a homogenous phantom should not be used in isolation because it is always necessary to obtain the required diagnostic information. The presented method is limited to X‐ray tubes with tungsten anodes. The implementation of this method requires only a simple template spreadsheet to run iterative calculations and a multimeter that can link to the actual spreadsheet. This linkage allows the measurement results to be automatically populated in the spreadsheet, as shown in Figure 3i. The template spreadsheet contains one sheet for calculation of the air kerma (Figure 3i) and one sheet for calculation of D_phantom_ (Figure 4). This template applies the coefficients from the parameterization, which are contained in separate sheets. One sheet (V) contains the coefficients associated with Equations (6) and (7) both for the target angles 10°, 12°, and 15° and for the parameterization of the spectra and the air kerma. Finally, the template contains a sheet (ε) with the coefficients associated with the parameterization of the energy imparted: 25 *a*_ij_ values for *T*_p_ (Equation 11), 24 *a*_ij_ values for *P*_ε_, and 24 *a*_ij_ values for *S*_ε_ (Equation 12), 16 *a*_ij_ values for Ψ/K_A_ (Equation 13), and 14 *a*_ij_ values for TF_Cu_ (Equation 15). These Excel sheets are available for free as Supporting Information in the journal.

## 5 CONCLUSION

We provide a method for computing the *ε* for conventional and fluoroscopic imaging for any PMMA thickness, filtration, kV, and geometry (SPD, SID). This calculation method is easily accessible in an Excel spreadsheet. The presented method has the advantages that absorbed doses from a large number of protocols and a wide range of patient sizes are easily obtained through a few measurements for each piece of X‐ray equipment. The corresponding results are provided immediately, and it is possible to perform calculations to investigate the absorbed dose impact from any changes in the exposure parameters, such as filtration and kV. The method allows dose comparison from protocols within and between the systems. In addition, it gives the user automatic feedback on the measurement results from a multimeter compared to the model. Hence, it is possible to immediately reveal whether the measurements are performed under the intended conditions. The presented method is useful as a part of QC of the X‐ray tube and appropriate D_phantom_ on periodic basis, and optimization of X‐ray systems.

## 6 CONFLICT OF INTEREST

None.
